# A comic based interactive digital intervention to enhance facilitation skills of nurse mentors in public facilities – results of a pilot intervention in Bihar, India

**DOI:** 10.1080/16549716.2023.2185365

**Published:** 2023-03-20

**Authors:** Rakesh Ghosh, Susanna R. Cohen, Nidhi Subramaniam, Seema Handu, Divya Vincent, Mikelle Lloyd, Kevin Thorn, Heidi Breeze Harris, Alisa Jenny, Dilys Walker

**Affiliations:** a Institute for Global Health Sciences, University of California, San Francisco, CA, USA; b LIFT Simulation Design Lab, Department of Obstetrics and Gynecology, University of Utah, Salt Lake City, Utah, USA; c Department of Simulation Implementation Research, PRONTO India Foundation, Lucknow, Uttar Pradesh, India; d PRONTO India Foundation, Patna, India; e PRONTO International, Seattle, WA, USA; f College of Nursing, University of Utah, Salt Lake City, UT, USA; g NuggetHead Studioz, LLC., Hernando, MS, USA; h School of Medicine, Department of Obstetrics, Gynecology and Reproductive Sciences, University of California, San Francisco, CA, USA

**Keywords:** Mass communication, simulation facilitation, labor and delivery care providers, digital storytelling, graphic medicine

## Abstract

**Background:**

Various trainings are designed to educate nurses to become simulation educators. However, there are no good strategies to sustain their learnings and keep them engaged. We developed a series of 10 interactive digital storytelling comic episodes ‘***The Adventures of Super Divya*** (SD)’ to strengthen simulation educator’s facilitation knowledge, skills, confidence, and engagement. This endline evaluation presents results on the change in knowledge after watching the episodes and retention of that knowledge over 10 months.

**Objectives:**

The objectives of this pilot study are to: 1) assess the change in knowledge between the baseline and post-episode surveys; and 2) understand the retention of knowledge between the post-episode and the endline survey.

**Methods:**

A human-centred design was used to create the episodes grounded in the lived experience of nurse simulation educators. The heroine of the comic is Divya, a ‘Super Facilitator’ and her nemesis is Professor Agni who wants to derail simulation as an educational strategy inside obstetric facilities. Professor Agni’s schemes represent real-life challenges; and SD uses effective facilitation and communication to overcome them. The episodes were shared with a group of nurse mentors (NM) and nurse mentor supervisors (NMS) who were trained to be champion simulation educators in their own facilities. To assess change in knowledge, we conducted a baseline, nine post-episode surveys and an endline survey between May 2021 and February 2022.

**Results:**

A total 110 NM and 50 NMS watched all 10 episodes and completed all of the surveys. On average, knowledge scores increased by 7–9 percentage points after watching the episodes. Comparison of survey responses obtained between 1 and 10 months suggest that the gain in knowledge was largely retained over time.

**Conclusions:**

Findings suggest that this interactive comic series was successful in a resource limited setting at engaging simulation educators and helped to maintain their facilitation knowledge over time.

## Introduction

Digital training in medicine is gaining foothold, albeit slowly, especially in low- and middle-income countries (LMICs). In this manuscript, we present a digital training modality with unique concepts for front-line intrapartum care mentors and providers in one of the poorest regions in South Asia, Bihar, India. Bihar is an eastern Indian state with neonatal mortality rate and maternal mortality ratio of 27 per 1,000 and 165 per 100,000 live births, respectively [1]. Despite having less than 10% of the country’s population, Bihar accounts for approximately 15% of neonatal deaths [2–4]. Primary health centres (PHCs) are the most common location of birth for the over 44 million women in the state. However, the PHCs are plagued with limited supplies and staffing with no obstetricians or paediatricians [1,3,5]. In 2015, the state Government of Bihar with a non-governmental organisation, CARE India, as the technical support unit, started implementing a statewide nurse-mentoring programme aimed at reducing maternal and neonatal deaths. Shortly thereafter, PRONTO International was invited to join the initiative and integrate its highly realistic in-situ simulation and team-training into the nurse-mentoring programme [6,7]. The resulting programme created a cadre of trained facility-level nurses who were encouraged to become champion simulation educators and independently conduct simulations as well as teamwork and communication (T&C) activities at their home facilities. As the cadre of champion simulation educators grew, a strategy was needed to sustain their learning and keep them engaged.

The LINQED (Leading Innovation in Quality-of-Care Education Development) team developed an innovative series of interactive digital comic episodes ‘***The Adventures of Super Divya*** (SD)’- a simulation educator superhero [8]. The goal of these storytelling comic episodes was to strengthen simulation educator’s facilitation knowledge, skills, confidence, and engagement. Comics and creative graphics have been used previously to communicate complex ideas about health [9]. The medium conveys nuanced concepts, explores emotional and social issues, and allows readers to develop a level of empathy beyond what can be achieved through textbooks [10]. Graphic and visualised contents have been shown to help readers retain information longer, improve comprehension, and understand complex concepts [9,11–13]. In formal health education settings, comics have been used among medical, nursing, and psychiatry students, to teach diverse topics from empathy to anatomy [11,14,15]. Although there has been some research into the use of digital comics in teaching, it is not known whether this method is effective with frontline nurses, specifically those individuals from rural areas where they may not have good access to the internet or have trouble navigating a smart phone. We chose to use the interactive graphics format because the topics we needed to cover were abstract and subtle, focused on the development of learners’ empathy skills, and facilitation of learning in resource-limited settings.

This endline evaluation presents results from the complete set of 10 SD comic episodes that were implemented with two cadres of simulation educators – nurse mentors (NM, frontline government staff nurses with a diploma in General or Auxiliary Nursing and Midwifery) and nurse mentor supervisors (NMS, nursing professionals in a mentoring role with a Bachelor’s or Master’s degree in Nursing, hired by CARE India). The objectives of this pilot study were to: 1) assess the change in knowledge between the baseline and post-episode surveys; and 2) understand the retention of knowledge between the post-episode and the endline survey.

## Methods

### Pilot study setting and participants

The idea of the SD intervention was conceived following the statewide AMANAT and AMANAT Jyoti programmes. AMANAT was a quality improvement initiative implemented by CARE India in collaboration with the Government of Bihar, in which among other components, visiting NMS conducted facility-based capacity building activities including in-situ highly realistic simulations. NMS mentored and supervised government facility-based nurses following a repeated cyclical pattern over approximately 8-months [16,17]. To promote sustainability, AMANAT was followed by the AMANAT Jyoti programme, in which NMS trained a group of about 800 government staff nurses, the NM from about 400 facilities in Bihar. These peer mentors then provided simulation trainings in their respective facilities [18]. A success of the programme was the creation of the educator infrastructure needed to achieve change at scale and sustain it over time in the state. In order to support the sustainability efforts, we began development of the SD digital storytelling video episodes. These digital, interactive episodes were targeted at the NMS and NM who had two prior simulation educator trainings (a 5-day introductory simulation and T&C facilitation training, and a 4-day advanced training). They were also provided with reviews of topics previously covered as part of the AMANAT and AMANAT Jyoti curriculum prior to the introduction of SD comics. The digital SD episodes were asynchronous, allowing individuals to progress at their own speed.

### Super Divya intervention

A human-centred design approach was used to create the storytelling episodes grounded in the lived experience of conducting simulation in the facilities in Bihar as well as to ensure the episode content and style were relatable [19]. The development of the episodes followed an iterative design, which included beta-testing, focus group discussions and a pilot survey before finalising and disseminating to the NM and NMS. After integrating feedback from the design process and from the initial qualitative discussions, 10 short (15–20 min) episodes in English and Hindi were designed by the LIFT Simulation Design Lab at the University of Utah in close collaboration with team members of PRONTO India Foundation and NuggetHead Studioz, LLC. Each episode focuses on a specific simulation facilitation topic with pre-defined objectives (Table 1). The Hindi version of the episodes was sent via WhatsApp to NMS and NM working in PHCs, District Hospitals, Sub-divisional Hospitals and Referral Hospitals in Bihar. Table 1 presents the objectives and timeline of each episode.

**Table 1.** Details of Super Divya episodes and distribution timeline.

Episode No.	Title	Learning Objectives	Date of distribution for Nurse Mentors	Date of distribution for Nurse Mentor Supervisors
Baseline Survey			May 2021	May 2021
1	Origin Story	Introduce the characters of the Super Divya comics Introduce the PRONTO Pouch Identify the elements of a good and bad simulation facilitation	May 2021	May 2021
2	Facilitation Secrets Part 1	Define a safe learning space List the components of a safe learning space and discuss how a safe learning space can be created	May 2021	May 2021
3	Facilitation Secrets Part 2	Introduce the energy scanner Introduce the concept of genuine self and discuss the genuine self-practices (be prepared, get support, ready…set…breathe and PRONTO pose)	June 2021	June 2021
4	Professor Agni Attacks the Pre-brief	List the four steps of a pre-brief Understand the importance of the four steps of a pre-brief	June 2021	June 2021
5	Super Divya Defends the Pre-brief	Understand the role of pre-brief in establishing a safe learning space Introduce facilitator spray	July 2021	July 2021
6	Teamwork and communication Part 1	Understand the importance of teamwork and communication techniques Introduce SBAR, check-back, thinking out loud, two challenge rule, call out Introduce the learning lens	August 2021	August 2021
7	Teamwork and communication Part 2	Practice recognising the SBAR communication technique	October 2021	October 2021
8	Super Divya and the Debrief huddle	Understand the importance of the debrief huddle Introduce learners to the empathy goggles	November 2021	November 2021
9	Super Divya decodes the Debrief	Understand the phases of the debrief How to ask open-ended questions How to protect confidentiality and the safe learning space Introduce the curiosity antenna	January 2022	January 2022
10	Guardians of the Debrief	Use of the empathy goggles Apply three stages of the debrief Practice tailoring debriefing questions based on participant’s feedback	February 2022	February 2022
Endline survey			February 2022	February 2022

The Adventures of Super Divya stories revolve around two characters: Divya, a ‘Super Facilitator’ and her nemesis, Professor Agni. Divya’s mission is to spread the magic of simulation and guide learners through practicing and understanding the nuanced elements of facilitation. Professor Agni’s goal is to derail Divya’s efforts by distracting simulation educators and leaners from meaningful engagement in simulation experiences and by taking away the safe learning space. Professor Agni’s schemes are designed to represent real-life challenges that nurse educators face in their daily work. SD displays leadership and uses effective facilitation and communication to overcome hurdles encountered while conducting simulations. Each episode contains interactive components and quizzes to keep learners actively engaged.

**Figure 1.**
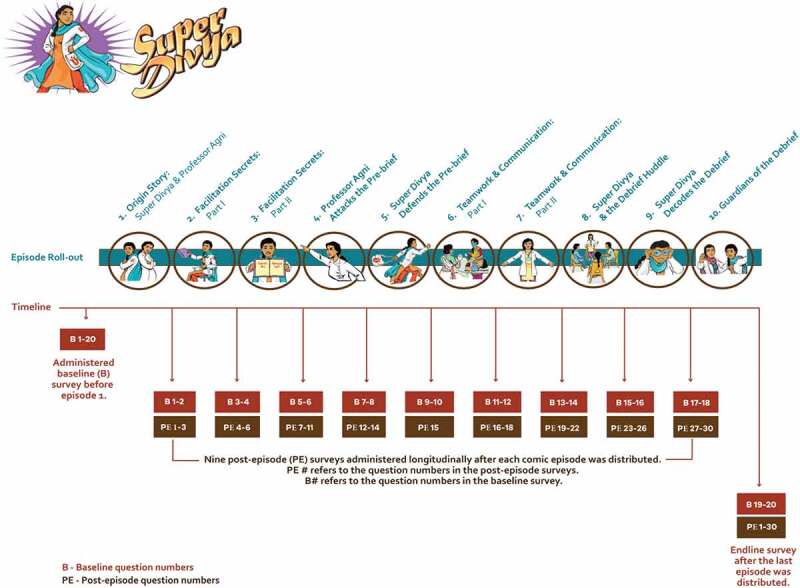
A description of the roll-out of the Super Divya episodes and the surveys to evaluate the digital comic intervention.

The SD comic intervention uses metaphors to convey abstract concepts. SD has a set of magical tools in her PRONTO Pouch, which represent advanced skills that educators can use to focus and manage their biases and distractions (Figure 1). For example: the *Facilitator spray* helps SD to recognise and name her own feelings, a critical step in self-reflection that is needed to prevent the educator from allowing their own issues into the learning space. The *Empathy goggles* help facilitators recognise clues from participant’s body language and facial expressions that help them to empathise with how the participants might be feeling at that moment. The *Curiosity antenna* helps facilitators to stay curious and reminds them to ask open-ended questions during the simulation debrief. The *Learning lens* helps take ‘snap shots’ during the simulation scenario. These snapshots enable the facilitators to capture key moments during the simulation, link them to the overall programme learning objectives and then use those as examples in the debrief. In addition, the episodes introduce a set of *Genuine self-practices*, which help in establishing a practice of mindfulness and help the educator stay focused and grounded in the face of challenges. Genuine self-practices include: *Ready-set-Breathe* in which SD recommends taking a moment to pause before facilitating an activity, and take three deep breaths; the *PRONTO pose* recommends assuming a ‘power’ pose of one’s choice and holding it for a minute while breathing deeply in and out, thus increasing confidence; *Be Prepared* minds the educator to prepare for the session by studying the curricular guide and to brainstorm solutions ahead of time for common problems encountered when conducting simulation; and *Get Support* helps to reach out to someone from work for support if you are struggling with any part of facilitation. The *Energy Scanner* is a 3 min guided meditation and body scan that the educators can use anytime they feel the need to recharge. All of these tools are used by SD to fight off a series of ‘bugs’ sent by Prof. Agni to derail the simulation. The bugs represent common educator and participant attitudes and distractions that pose a challenge to even the most experienced facilitator. Some of these bugs include *fluster, fear, ego, disinterest, self-doubt, defensiveness, and frustration*. The use of the bug imagery conveys the message that participants are not inherently difficult rather they have been *bitten* and are temporarily inflicted by the negative attitude that prevents their full engagement in the learning.

Baseline survey before any comic episode were distributed. B# refers to the question numbers in the baseline survey.

A total of nine post-episode surveys administered longitudinally after each comic episode was distributed. PE # refers to the question numbers in the post-episode surveys.

Endline survey after the last comic episode was distributed.

### Survey description and sample selection

Web surveys in English and Hindi were designed using first Qualtrics and later Google (the shift was to overcome accessibility issues identified early on in the study). Prior to distributing the first episode, participants completed a baseline survey containing 20 true/false questions that covered topics from all 10 episodes (Figure 1, Supplementary Table S1). Participants then received a short post-episode survey after they watched each episode (Figure 1). Post-episode surveys repeated two questions (shown as B# in Figure 1) from the baseline survey and two to four new in-depth questions on the topic of the episode (shown as PE# in Figure 1). After the last episode, an endline survey was conducted that repeated all the new in-depth questions (i.e. PE 1–37 in Figure 1) from the post-episode surveys. The endline survey did not repeat the baseline survey questions because they were already administered twice and provided necessary data to measure change. Thus, we had the baseline survey, nine brief post-episode surveys and the endline survey. Across all surveys, each question was asked twice. One set of pre-post questions (B# in Figure 1) allowed us to assess change in knowledge, after watching the episodes. The second set of pre-post questions (PE# in Figure 1) allowed us to assess retention in knowledge, over 1–10 months. Nurses completed the surveys on their smart mobile devices. All of the questions were mandatory. The survey offered true/false, multiple choice, or select all that apply responses, as appropriate. The correct answers to each question appeared after the responses were submitted.

A convenient sample of 148 NM out of 685 were selected based on their availability and willingness to participate. The selected NM were oriented by their respective NMS in their facility on how to access the SD digital episodes. A sample survey link was also shared. They were asked to watch a SD trailer on their smart phone and respond to the sample survey, to demonstrate that they grasped the necessary steps. All 77 NMS participated in the pilot intervention.

### Statistical analysis

We matched the responses of individual NM and NMS across the baseline, post-episode and endline surveys. Score for an individual was the number of correct responses divided by the total number of questions multiplied by 100. For each participant, we generated their baseline score, two sets of post-episode survey scores and the endline score. The baseline score was generated using the responses to the 20 questions asked in the baseline survey (Figure 1). The first set of post-episode survey scores (Post-episode score 1) was generated using the post responses to the 20 baseline questions (shown as B# in Figure 1) episode. The second set of post-episode survey scores (Post-episode score 2) was generated using the responses to the 30 new in-depth questions (not part of baseline) asked in the post-episode survey (shown as PE# in Figure 1). Finally, the endline score was generated using the repeat responses to 30 new in-depth questions repeated in the endline survey. We compared the baseline score with the post-episode score 1 to evaluate knowledge gained after they watched the SD episodes. Next, we compared the Post-episode score 2 with the endline score to evaluate knowledge retained by the end of the last episode. In other words, participants’ responses were compared for the 20 baseline questions that were repeated in the post-episode surveys to understand immediate improvement, while the comparison of the 30 post-episode survey questions that were repeated in the endline helped to understand retention of knowledge. The individual scores are reported in four categories: 100%, 99–80%, 79–60% and below 60%. In addition, we present the mean scores with the standard deviation. Analyses were conducted and reported separately for NM and NMS. Excel and Stata 17.0 were used for the analysis, and SQUIRE guidelines were followed for reporting in this manuscript [20].

## Results

A total 110 NM (out of 148, 74%) and 50 NMS (out of 77, 65%) participants completed all of the surveys and were included in this analysis. The vast majority (83%) of the NM had completed a diploma in general nursing and midwifery, while a majority (78%) of the NMS completed bachelor’s degrees (Table 2). About 94% of NM and 66% of NMS had participated either in the AMANAT or AMANAT Jyoti nurse-mentoring programmes. 80% of the NM and all NMS were trained by PRONTO International on facilitating simulation and T&C activities either through in-person or virtual training prior to inclusion in this pilot study. About half (48%) of the NM and two-thirds (66%) of the NMS facilitated at least one simulation per month prior to COVID-19 related interruptions (Table 2).

**Table 2.** Characteristics of the nurse mentors and nurse mentor supervisors, the participants of the Super Divya surveys implemented from May 2021 to February 2022.

	Nurse Mentors (*N* = 110)	Nurse Mentor Supervisors (*N* = 50)
	% (n)	% (n)
*Qualification*		
General nursing and midwifery	83 (91)	N/A
Auxiliary nursing and midwifery	16 (18)	N/A
Post diploma in nursing	N/A	10 (5)
Bachelor’s degree in nursing	1 (1)	78 (39)
Master’s degree in nursing	0 (0)	12 (6)
*Participated in the AMANAT or AMANAT Jyoti Programs*		
Yes	94 (103)	66 (33)
No	6 (7)	34 (17)
*Previously trained by PRONTO*		
Yes	80 (88)	100 (50)
No	20 (22)	0 (0)
*Number of simulations conducted monthly prior to COVID-19 interruptions*		
0	52 (57)	34 (17)
1–3	46 (51)	54 (27)
4–6	1 (1)	10 (5)
7–9	0 (0)	0 (0)
10 or more	1 (1)	2 (1)

Overall, the individual scores showed improvement between baseline and post-episode survey score 1 (Table 3). A higher proportion of NM scored in the 80–99% category in the post-episode survey score 1 (64%) than at baseline (30%). Simultaneously, a smaller proportion scored in the 60–79% category in the post-episode survey score 1 (32%) than at baseline (64%). Similarly, the NMS scores also showed an improvement (Table 3). For example, none of the NMS achieved 100% at baseline, whereas in the post-episode survey score 1, 8% of the participants scored 100%. Moreover, the proportion of NMS who scored in the 80–99% category increased from 40% at baseline to 72% in the post-episode survey score 1, whereas those in the 60–79% category decreased from 60% at baseline to 20% at post-episode survey score 1. The average scores between baseline and post-episode surveys score 1 increased by about 9 and 7 percentage points for the NM and NMS, respectively (Table 3). Scores of individual questions are presented in Supplementary table S2.

**Table 3.** Count and percentage of participants who answered the knowledge questions correctly in the baseline, post-episode and endline surveys, separately for the Nurse Mentors and Nurse Mentor Supervisors.

Score categories	Nurse Mentors (*N* = 110)				Nurse Mentor Supervisors (*N* = 50)			
B 1–20	Baseline		Post-episode score 1*		Baseline*		Post-episode score 1*	
	*n*	%	*n*	%	*n*	%	*n*	%
100	0	0	1	1	0	0	4	8
80–99	33	30	70	64	20	40	36	72
60–79	70	64	35	32	30	60	10	20
<60	7	6	4	4	0	0	0	0
Mean (SD)	71% (10)		80% (10)		78% (9)		85% (9)	
	Post-episode score 2^#^		Endline^!^		Post-episode score 2^#^		Endline^!^	
PE 1–30	n	%	n	%	n	%	n	%
100	10	9	0	0	0	0	5	10
80–99	30	27	50	45	38	76	33	66
60–79	53	48	45	41	11	22	10	20
<60	17	15	15	14	1	2	2	4
Mean (SD)	73% (15)		75% (12)		86% (10)		84% (12)	

*Post-episode score 1 was generated using the 20 baseline questions that were repeated in the post-episode surveys (shown as B # in Figure 1).

^#^Post-episode score 2 was generated using the 30 new in-depth questions that were not part of the baseline survey (shown as PE # in Figure 1).

^!^Endline score was generated using the 30 new in-depth post-episode questions that were repeated in the endline survey.

A comparison of the post-episode survey score 2 with the endline survey score indicates that both cadres largely retained knowledge over a period of maximum 10 months (Table 3). The proportion of NM who were in the 100% score category decreased from 9% in the post-episode survey score 2 to 0% at endline. However, in the 80–99% score category an upward trend was observed from 27% in the post-episode survey score 2 to 45% at endline. For the NMS, an upward trend was observed in the 100% score category as none of NMS achieved that score in the post-episode survey score 2, whereas in the endline survey 10% achieved that score. The average scores for the two timepoints were largely similar for both cadres (Table 3). When the questions that were asked both at the post-episode survey and in the endline survey were examined individually, we see less than 10% decrease in score between the two timepoints for all questions (Supplementary Table S3).

## Discussion

In this pilot study, we explored whether The Adventures of Super Divya, an interactive digital comic series, could sustain and improve simulation educator knowledge among front-line intrapartum care nurses in Bihar, India. A comparison of results from surveys conducted at several timepoints suggests an improvement in knowledge. More importantly, the knowledge gained from the interactive comics was largely retained over a maximum of 10 months. Note, the maximum gap in time was 10 months between the 1^st^ and the 10^th^ episode, while the minimum gap was 1 month between the 9^th^ and the 10^th^ episode. These findings indicate that the comic episodes appear to be successful in improving and retaining knowledge pertaining to best-practices for successfully conducting simulation training (e.g. creating a safe learning environment, conducting a pre-brief, facilitating a debrief and understanding T&C techniques [21]).

A few gaps emerged that point towards the need for additional reinforcement for some of the novel and relatively complex concepts of a simulation, specifically related to conducting the pre-brief and phases of a debrief. While some of the new concepts (e.g. Genuine self-practice, PRONTO pose, Facilitator spray, Fluster bugs) were well-understood by the nurse educators, others (e.g. Empathy goggles, Curiosity antenna, Learning lens) require further investigation to understand whether the concepts behind the ‘tools’ were not grasped or the lack of change in scores was due to the manner of assessment. Like the specific concepts, individual episodes may have been at varying levels of difficulty, because performance across episodes varied, suggesting improvement and/or retention of knowledge may not have been uniform. For example, in the comparison of the post-episode score 2 with endline, we found both cadres registered best performance in the Origin Story and Facilitation Secrets Part 2 episodes and worst performances in the Professor Agni Attacks the Pre-brief episode. Nevertheless, this study demonstrates that using digital comics, basic simulation and T&C facilitation concepts can be maintained and nuanced concepts can be introduced, even in this low-resource setting. To the best of our knowledge, this interactive comic series is the first report of using this modality to strengthen knowledge about complex facilitation concepts for frontline nurse educators in a resource-limited setting.

It has been suggested that the use of comics in healthcare education can provide an alternative mode for reflection, (compared to reflection through writing or verbal communication), foster thinking, help linking theory with practice, and simplify communication by adding humour [22]. Comics can also offer a safe space for expression [23], including sharing life experiences, though all of these instances are in the Western settings [24]. Recently, a report presented the perception of medical and nursing students about the use of comics in health education, in New Delhi, India. While only one-fifth reported having heard about graphic education, three-quarters wanted comics to be used in health training [25]. With the rapid spread of internet and mobile digital platforms, access to the digital environment is getting easier, even in the resource-limited settings. This study provides a proof of concept that digital comics can be effectively used for training frontline health providers.

The intervention was strengthened by the creativity and innovation of the designers through the use of a super hero nurse. Each episode had specific learning objectives, laid out at the outset of each episode. The graphics were attractive and aimed to be relatable because they were designed using images of local nurses and the settings reflected a health centre in a LMIC. The story line was appealing because of the depiction of clashes between a protagonist and an antagonist, in a classic good-vs-evil struggle. The nurse educators felt connected because the challenges depicted in the episodes were among those the nurses routinely encounter. Use of local language (Hindi) made the episodes culturally appropriate and easy to understand, though an English version is also available. Lastly, the design of the knowledge assessment surveys was similar to a case-crossover study where the participants served as their own controls.

There were several challenges, including difficulties accessing the episodes and registering responses to the online surveys, due to intermittent connectivity issues encountered by a small number of participants. To alleviate these problems, PRONTO provided free data to watch the episodes and to complete the surveys. There was no way to ensure that nurses responded to post-episode surveys after viewing the episodes; some nurses may have responded to surveys without watching the SD episodes. It is also possible that varied participation in previous PRONTO and/or AMANAT trainings as well as years of experience as a simulation educator may have influenced the results. We also faced challenges with the roll-out of the episodes and retention of participants over time. Initially, we distributed the episodes to 148 NM and 77 NMS, of which 110 NM and 50 NMS were retained. The loss of NM was due to reassignment to COVID-19 services or due to transfer to other departments within the same or another facility. The loss to follow-up may have introduced bias in our results. Due to constraints beyond the control of the study team, such as staff turnover or transfer to a different district during the AMANAT programme, we lost some NMS from this pilot project. Another limitation was not having a control group and the intervention was not assigned in a randomised manner, limiting the interpretational ability of the results. Finally, the initial selection of the NM and NMS was based on their availability, workload and willingness to participate in the SD intervention. Hence, results may or may not be generalisable to all NM and NMS.

## Conclusion

Overall, the Adventures of Super Divya episodes appear to have helped improve and sustain knowledge for two cadres of simulation educators in Bihar. The results from this pilot study are encouraging, especially during a global pandemic when digital learning and training became imperative, not to mention the cost savings in using this digital environment. These findings suggest The Adventures of Super Divya interactive comic series is an innovative training tool for maintaining simulation educator knowledge and skills in resource-limited settings. Effective training and engagement of educators has the potential to improve the quality of obstetric and neonatal care and ultimately patient outcomes.

## Supplementary Material

Supplemental Material

## Paper context

Comics have been successfully used to teach clinicians a range of topics from empathy to anatomy. However, there is scant evidence about their use with frontline nurses in low-resource settings. To our knowledge, this is the first study that demonstrates use of interactive digital storytelling comics with labour and delivery care nurses in the public health facilities in Bihar, India. The comics were popular and appear to have improved knowledge of this group.

## References

[1] NITI [National Institution for Transforming India]. Aayog, Government of India. Handbook of State Statistics. New Delhi: Government of India; 2018 [cited 2019 Mar 25]. Available from: http://niti.gov.in/state-statistics

[2] Godinho MA, Murthy S, Lakiang T, Puranik A, Nair SN. Mapping neonatal mortality in India: a closer look. Indian J Community Med. 2017;42:234. 29184326 10.4103/ijcm.IJCM_327_16 PMC5682725

[3] Ministry of Health and Family Welfare; Government of India. Rural health mission statistics 2020-21. New Delhi: Ministry of Health and Family Welfare, Statistics Division; 2020-2021.

[4] Ministry of Health and Family Welfare; Government of India. India Newborn Action Plan New Delhi, India: National Health Mission; 2022 [cited 2022 Aug 17]. Available from: https://nhm.gov.in/index4.php?lang=1&level=0&linkid=153&lid=174

[5] Vail B, Morgan M, Spindler H, Christmas A, Cohen S, Walker D. The power of practice: simulation training improving the quality of neonatal resuscitation skills in Bihar, India. BMC Pediatr. 2018;18. DOI:10.1186/s12887-018-1254-0 PMC6122678 30176831

[6] Das A, Nawal D, Singh MK, Karthick M, Pahwa P, Shah MB, et al. Evaluation of the mobile nurse training [MNT] intervention–a step towards improvement in intrapartum practices in Bihar, India. Bmc Pregnancy Childbirth. 2017;17:266. 28835213 10.1186/s12884-017-1452-z PMC5569501

[7] Das A, Nawal D, Singh MK, Karthick M, Pahwa P, Shah MB, et al. Impact of a nursing skill-improvement intervention on newborn-specific delivery practices: an experience from Bihar, India. Birth. 2016;43:328–9. 27321470 10.1111/birt.12239

[8] Kalra A, Subramaniam N, Longkumer O, Siju M, Jose LS, Srivastava R, et al. Super Divya, an interactive digital storytelling instructional comic series to sustain facilitation skills of labor and delivery nurse mentors in Bihar, India—a pilot study. Int J Environ Res Public Health. 2022;19:2675. 35270366 10.3390/ijerph19052675 PMC8910046

[9] Green MJ, Myers KR. Graphic medicine: use of comics in medical education and patient care. BMJ. 2010;340. DOI:10.1136/bmj.c863 20200064

[10] Hanson A, Drendel AL, Ashwal G, Thomas A. The feasibility of utilizing a comic for education in the emergency department setting. Health Commun. 2017;32:529–532. 27540632 10.1080/10410236.2016.1211076

[11] Kim J, Chung MS, Jang HG, Chung BS. The use of educational comics in learning anatomy among multiple student groups. Anat Sci Educ. 2017;10:79–86. 27233080 10.1002/ase.1619

[12] Liu J. Effects of comic strips on L2 learners’ reading comprehension. TESOL Quart. 2004;38:225–243.

[13] Rapp DN. Comic books’ latest plot twist: enhancing literacy instruction. Phi Delta Kappan. 2011;93:64–67.

[14] Tsao P, Yu CH. “There’s no billing code for empathy”-Animated comics remind medical students of empathy: a qualitative study. BMC Med Educ. 2016;16:1–8. 27520824 10.1186/s12909-016-0724-z PMC4983096

[15] Joshi A, Hillwig-Garcia J, Joshi M, Lehman E, Khan A, Llorente A, et al. Comics as an educational tool on a clinical clerkship. Acad Psychiatry. 2019;43:290–293. 30607894 10.1007/s40596-018-1016-1

[16] Ghosh R, Spindler H, Dyer J, Christmas A, Cohen S, Das A, et al. Simulation and team training embedded nurse mentoring program and improvement in intrapartum and newborn care in a low-resource setting in India. J Glob Health. 2019;10. DOI:10.7189/jogh.10.0201010 PMC7759018 33425334

[17] Ghosh R, Spindler H, Morgan M, Cohen S, Begum N, Gore A, et al. Diagnosis and management of postpartum hemorrhage and intrapartum asphyxia in a quality improvement initiative using nurse-mentoring and simulation in Bihar, India. PLoS ONE. 2019;14:e0216654. 31276503 10.1371/journal.pone.0216654 PMC6611567

[18] Koon AD, Hoover J, Sonthalia S, Rosser E, Gore A, Rao KD. In-service nurse mentoring in 2020, the year of the nurse and the midwife: learning from Bihar, India. Global Health Action. 2020;13:1823101. 33023408 10.1080/16549716.2020.1823101 PMC7580717

[19] Lloyd D. Evaluating human-centered approaches for geovisualization. London: City University London; 2009.

[20] Davidoff F, Batalden P, Stevens D, Ogrinc G, Mooney SE. Publication guidelines for quality improvement studies in health care: evolution of the SQUIRE project. BMJ. 2009;338:a3152. 19153129 10.1136/bmj.a3152 PMC2769030

[21] Standards Committee INACSL, Persico L, Belle A, DiGregorio H, Wilson-Keates B, Shelton C. Healthcare simulation standards of best practiceTM facilitation. Clini Simul Nursing. 2021;58:22–26.

[22] Whiting J. Comics as reflection: in opposition to formulaic recipes for reflective processes. Perm J. 2020;24. DOI:10.7812/TPP/19.134 PMC6907911 31852060

[23] Go Home MJ. Med student: comics as visual media for students’ traumatic medical education experiences. AMA J Ethics. 2018;20:141–147. 29460766 10.1001/journalofethics.2018.20.2.ecas2-1802

[24] Green MJ. Comics and medicine: peering into the process of professional identity formation. Acad Med. 2015;90:774–779. 25853686 10.1097/ACM.0000000000000703

[25] Anand T, Kishore J, Ingle GK, Grover S. Perception about use of comics in medical and nursing education among students in health professions’ schools in New Delhi. Educ Health. 2018;31:125. 10.4103/efh.EfH_298_15 30531056

