# Supplementary material for: A comic based interactive digital intervention to enhance facilitation skills of nurse mentors in public facilities – results of a pilot intervention in Bihar, India
Source: Glob Health Action. 2023 Mar 20;16(1):2185365. doi: 10.1080/16549716.2023.2185365 (PMC10035940; doi:10.1080/16549716.2023.2185365)
Supplement: Supplemental Material [file ZGHA_A_2185365_SM0545.docx]

**Super Divya Manuscript Supplementary Information**

**A comic based interactive digital intervention to enhance facilitation skills of nurse mentors in public facilities – results of a pilot intervention in Bihar, India**

Rakesh Ghosh^1^, Susanna R. Cohen^2^, Nidhi Subramaniam^3^, Seema Handu^4^, Divya Vincent^3^, Mikelle Lloyd^5^, Kevin Thorn^6^, Heidi Breeze-Harris^4^, Alisa Jenny^1^, Dilys Walker^1,7^

^1^ Institute for Global Health Sciences, University of California, San Francisco, CA 94158, USA

^2^ LIFT Simulation Design Lab, Department of Obstetrics and Gynecology, University of Utah, Salt Lake City, Utah, UT 84132, USA

^3^ PRONTO India Foundation, Lucknow 226001, Uttar Pradesh, and Patna 800025, Bihar, India

^4^ PRONTO International, 5419 Greenwood Ave N Seattle, WA 98103, USA

^5^ College of Nursing, University of Utah, Salt Lake City, UT 84112, USA

^6^ NuggetHead Studioz, LLC., 1862 Gracie Road, Hernando, MS 38632, USA

^7^ School of Medicine, Department of Obstetrics, Gynecology and Reproductive Sciences, University of California, San Francisco, CA 94158, USA

***Corresponding author:** Rakesh Ghosh, Institute for Global Health Sciences, University of California San Francisco, 550 16th St, San Francisco, California, 94158, USA. Email: [Rakesh.Ghosh@ucsf.edu](mailto:Rakesh.Ghosh@ucsf.edu)

**SUPPLEMENTARY INFORMATION**

**Supplemental Table 1.** Episode specific questions used in the baseline, post-episode and endline surveys to assess knowledge.

| **Episode** | **Survey question** | **Baseline survey** | **Post episode surveys** | **Endline survey** |
| --- | --- | --- | --- | --- |
| Episode 1- Origin | 1. A facilitator knows all the answer to participants' questions. | ✓* | ✓ |  |
|  | 1. A facilitator should stop the simulation when a nurse misses a clinical management step. | ✓ | ✓ |  |
|  | 1. A facilitator makes everyone feel comfortable and welcome. |  | ✓ | ✓ |
|  | 1. A facilitator encourages participants to learn from errors made in simulation. |  | ✓ | ✓ |
|  | 1. A facilitator points out only errors and ignores the good things. |  | ✓ | ✓ |
| Episode 2- Facilitation secrets part 1 | 1. Greeting nurses when they arrive at the training is an important step in creating safe learning space. | ✓ | ✓ |  |
|  | 1. In a safe learning space nurses feel supported and open to learning. | ✓ | ✓ |  |
|  | 1. The rules of simulation are only reviewed the first time you do simulation with a group of learners. |  | ✓ | ✓ |
|  | 1. Super Divya suggests having a notebook to write down observations during the simulation scenario. |  | ✓ | ✓ |
|  | 1. To create a safe learning space, the facilitator should locate and dispose the containers of hazardous biomedical waste for all participants. |  | ✓ | ✓ |
| Episode 3- Facilitation secrets part 2 | 1. To bring my genuine self, I need to avoid challenging situations. | ✓ | ✓ |  |
|  | 1. The energy scanner is a tool that Super Divya uses to measure the energy of others in the room. | ✓ | ✓ |  |
|  | 1. A facilitator can influence how participants feel during the simulation |  | ✓ | ✓ |
|  | 1. According to Super Divya, the most important thing a facilitator brings to a simulation is her authority. |  | ✓ | ✓ |
|  | 1. The concept of the genuine self means to work independently and solve my own problems. |  | ✓ | ✓ |
|  | 1. The “Genuine Self” includes the facilitator’s listening skills, empathy, compassion and curiosity. |  | ✓ | ✓ |
|  | 1. To bring my genuine self to a training, I need to know the correct answer for all the questions the nurses ask. |  | ✓ | ✓ |
| Episode 4- Professor Agni attacks the Pre-brief | 1. During the pre-brief, the facilitator should remind participants how to manage medical complications. | ✓ | ✓ |  |
|  | 1. During the pre-brief, the facilitator should allow providers time to review the simulation area. | ✓ | ✓ |  |
|  | 1. The pre-brief is part of making a safe learning space. |  | ✓ | ✓ |
|  | 1. A participant can ask the facilitator for clinical help at any time during a simulation. |  | ✓ | ✓ |
|  | 1. Identify the 4 components of the pre-brief |  | ✓ | ✓ |
| Episode 5- Super Divya defends the Pre-brief | 1. A genuine self-practice can help a facilitator calm her mind to feel centered. | ✓ | ✓ |  |
|  | 1. For a successful simulation training, the facilitators need to ignore their own emotions. | ✓ | ✓ |  |
|  | 1. The facilitator spray is an imaginary tool that can be used to: |  | ✓ | ✓ |
| Episode 6- Teamwork and communication part 1 | 1. Clinical knowledge is a behavioral objective. | ✓ | ✓ |  |
|  | 1. If there are communication issues in the simulation, the facilitator should bring them up in the debrief. | ✓ | ✓ |  |
|  | 1. The learning lens is a helpful tool when the power/electricity goes off. |  | ✓ | ✓ |
|  | 1. Different communication techniques such as two challenge rule or check back could prevent you from making a clinical error during an emergency |  | ✓ | ✓ |
|  | 1. She arrives at the facility with her husband and was taken to the triage room. What is the very first thing you should do when you meet Ruchi? |  | ✓ | ✓ |
| Episode 7- Teamwork and communication part 2 | 1. An SBAR is given when a new nurse or doctor enters the clinical scenario to provide details of the patient's medical history. | ✓ | ✓ |  |
|  | 1. Paying attention during the simulation for examples of the communication techniques can help me prepare for the debrief. | ✓ | ✓ |  |
|  | 1. What does the communication technique SBAR stand for? |  | ✓ | ✓ |
|  | 1. Provider 2 arrives to help Provider 1 with neonatal resuscitation. Provider 1 says, “Priya is gravida 2, para 1, with four antenatal care visits. She has had a six-hour labor. Her baby was just born and her husband is in the waiting area. Did Provider 1 demonstrate all four components of SBAR? |  | ✓ | ✓ |
|  | 1. The fluster bugs that Professor Agni unleashed were meant to... |  | ✓ | ✓ |
|  | 1. During a simulation there is a lot of action going on at the same time. How do you focus on the important moments? |  | ✓ | ✓ |
| Episode 8- Introducing the Debrief | 1. Empathy means to understand one's and other's feelings. | ✓ | ✓ |  |
|  | 1. One of the goals of the debrief huddle is to decide which nurses should speak up first. | ✓ | ✓ |  |
|  | 1. When should a facilitator conduct a debrief huddle? |  | ✓ | ✓ |
|  | 1. The debrief huddle helps in: |  | ✓ | ✓ |
|  | 1. What should a simulation facilitator do during the release moment? |  | ✓ | ✓ |
|  | 1. Empathy goggles help a facilitator identify the nurses who know the right answers during the debrief. |  | ✓ | ✓ |
| Episode 9- Super Divya decodes the Debrief | 1. The debrief has 3 phases: description, recommendation, and application. | ✓ | ✓ |  |
|  | 1. Open-ended questions are helpful in the debrief to understand the nurses’ thoughts and feelings. | ✓ | ✓ |  |
|  | 1. Why is the analysis phase of a debrief the longest? |  | ✓ | ✓ |
|  | 1. What happens when a facilitator lets their ego get in the way of debriefing? |  | ✓ | ✓ |
|  | 1. What should the participants do during the application phase? |  | ✓ | ✓ |
|  | 1. How does curiosity help a facilitator? |  | ✓ | ✓ |
| Episode 10- The Epic Battle over the Debrief | 1. When participants show disinterest during the debrief, the facilitator should ignore them. | ✓ | ✓ |  |
|  | 1. If everything went well during the simulation, there's no need to have a debrief. | ✓ | ✓ |  |
|  | 1. A facilitator controls how actively the participants engage in a debriefing session |  |  | ✓ |
|  | 1. When a participant who has made an error gets defensive during a debrief, raises her voice, and resists self-reflection, what can an experienced (super) facilitator do to re-engage her in the learning? |  |  | ✓ |
|  | 1. During the debrief, if a participant is extremely nervous, worried about sharing, and looking down at the ground, what feeling might she be experiencing? |  |  | ✓ |
|  | 1. When a participant says something like, “I have ten years of experience, I have always given medications in this order, and it always works.” Which feeling do you think she might be experiencing? |  |  | ✓ |
|  | 1. If the nurses are not interested in participating in the simulation, what can the facilitator do to encourage them: |  |  | ✓ |

* The tick signifies that the question was used in that survey.

**Supplemental Table 2**. Performance of the Nurse Mentor Supervisors (NMS) and Nurse Mentors (NM) on the individual questions in the baseline and in the post episode (immediately after watching a comic episode).

| **Episode** | **Survey question** | **Staff category** | **Baseline survey score** | **Post episode surveys** |
| --- | --- | --- | --- | --- |
|  |  |  | **% (n)** | **% (n)** |
| **Episode 1- Origin** | 1. A facilitator knows all the answer to participants' questions. | NMS | 46 (23) | 56 (28) |
|  |  | NM | 13 (14) | 66 (73) |
|  | 2. A facilitator should stop the simulation when a nurse misses a clinical management step. | NMS | 90 (45) | 98 (49) |
|  |  | NM | 75 (83) | 92 (101) |
| **Episode 2- Facilitation secrets part 1** | 3. Greeting nurses when they arrive at the training is an important step in creating safe learning space. | NMS | 98 (49) | 100 (50) |
|  |  | NM | 97 (107) | 97 (107) |
|  | 4. In a safe learning space nurses feel supported and open to learning. | NMS | 100 (50) | 100 (50) |
|  |  | NM | 99 (109) | 99 (109) |
| **Episode 3- Facilitation secrets part 2** | 5. To bring my genuine self, I need to avoid challenging situations. | NMS | 96 (48) | 92 (46) |
|  |  | NM | 68 (75) | 70 (77) |
|  | 6. The energy scanner is a tool that Super Divya uses to measure the energy of others in the room. | NMS | 52 (26) | 84 (42) |
|  |  | NM | 31 (34) | 49 (54) |
| **Episode 4- Professor Agni attacks the Pre-brief** | 7. During the pre-brief, the facilitator should remind participants how to manage medical complications. | NMS | 84 (42) | 88 (44) |
|  |  | NM | 28 (31) | 87 (96) |
|  | 8. During the pre-brief, the facilitator should allow providers time to review the simulation area. | NMS | 88 (44) | 94 (47) |
|  |  | NM | 95 (105) | 97 (107) |
| **Episode 5- Super Divya defends the Pre-brief** | 9. A genuine self-practice can help a facilitator calm her mind to feel centered. | NMS | 98 (49) | 98 (49) |
|  |  | NM | 96 (106) | 96 (106) |
|  | 10. For a successful simulation training, the facilitators need to ignore their own emotions. | NMS | 44 (22) | 60 (30) |
|  |  | NM | 43 (47) | 64 (70) |
| **Episode 6- Teamwork and communication part 1** | 11. Clinical knowledge is a behavioral objective. | NMS | 52 (26) | 86 (43) |
|  |  | NM | 54 (59) | 54 (59) |
|  | 12. If there are communication issues in the simulation, the facilitator should bring them up in the debrief. | NMS | 100 (50) | 100 (50) |
|  |  | NM | 94 (103) | 94 (103) |
| **Episode 7- Teamwork and communication part 2** | 13. An SBAR is given when a new nurse or doctor enters the clinical scenario to provide details of the patient's medical history. | NMS | 8 (4) | 20 (10) |
|  |  | NM | 41 (45) | 41 (45) |
|  | 14. Paying attention during the simulation for examples of the communication techniques can help me prepare for the debrief. | NMS | 98 (49) | 100 (50) |
|  |  | NM | 95 (104) | 95 (104) |
| **Episode 8- Introducing the Debrief** | 15. Empathy means to understand one's and other's feelings. | NMS | 80 (40) | 94 (47) |
|  |  | NM | 95 (104) | 95 (104) |
|  | 16. One of the goals of the debrief huddle is to decide which nurses should speak up first. | NMS | 60 (30) | 74 (37) |
|  |  | NM | 70 (77) | 70 (77) |
| **Episode 9- Super Divya decodes the Debrief** | 17. The debrief has 3 phases: description, recommendation, and application. | NMS | 16 (8) | 60 (30) |
|  |  | NM | 47 (52) | 47 (52) |
|  | 18. Open-ended questions are helpful in the debrief to understand the nurses’ thoughts and feelings. | NMS | 94 (47) | 100 (50) |
|  |  | NM | 100 (110) | 100 (110) |
| **Episode 10- The Epic Battle over the Debrief** | 19. When participants show disinterest during the debrief, the facilitator should ignore them. | NMS | 94 (47) | 96 (48) |
|  |  | NM | 88 (97) | 95 (104) |
|  | 20. If everything went well during the simulation, there's no need to have a debrief. | NMS | 98 (49) | 98 (49) |
|  |  | NM | 85 (94) | 92 (101) |

**Supplemental Table 3**. Performance of the Nurse Mentor Supervisors (NMS) and Nurse Mentors (NM) on the individual questions in the post episode (immediately after watching a comic episode) and in the endline survey (after the 10^th^ comic episode).

| **Episode** | **Survey question** | **Staff category** | **Post episode surveys** | **Endline survey score** |
| --- | --- | --- | --- | --- |
|  |  |  | **% (n)** | **% (n)** |
| **Episode 1- Origin story** | 1. A facilitator makes everyone feel comfortable and welcome | NMS | 100 (50) | 100 (50) |
|  |  | NM | 99 (109) | 98 (108) |
|  | 2. A facilitator encourages participants to learn from errors made in simulation | NMS | 98 (49) | 100 (50) |
|  |  | NM | 95 (105) | 89 (98) |
|  | 3. A facilitator points out only errors and ignores the good things | NMS | 96 (48) | 100 (50) |
|  |  | NM | 96 (106) | 98 (108) |
| **Episode 2- Facilitation Secrets Part 1** | 4. The rules of simulation are only reviewed the first time you do simulation with a group of learners | NMS | 94 (47) | 94 (47) |
|  |  | NM | 91 (100) | 96 (106) |
|  | 5. Super Divya suggests having a notebook to write down observations during the simulation scenario | NMS | 96 (48) | 84 (42) |
|  |  | NM | 95 (105) | 78 (86) |
|  | 6. To create a safe learning space, the facilitator should locate and dispose the containers of hazardous biomedical waste for all participants. | NMS | 58 (29) | 62 (31) |
|  |  | NM | 41 (45) | 66 (73) |
| **Episode 3- Facilitation Secrets Part 2** | 7. A facilitator can influence how participants feel during the simulation | NMS | 98 (49) | 100 (50) |
|  |  | NM | 94 (103) | 89 (98) |
|  | 8. According to Super Divya, the most important thing a facilitator brings to a simulation is her authority. | NMS | 82 (41) | 86 (43) |
|  |  | NM | 61 (67) | 79 (87) |
|  | 9. The concept of the genuine self means to work independently and solve my own problems. | NMS | 62 (31) | 64 (32) |
|  |  | NM | 31 (34) | 56 (62) |
|  | 10. The “Genuine Self” includes the facilitator’s listening skills, empathy, compassion and curiosity. | NMS | 96 (48) | 94 (47) |
|  |  | NM | 100 (110) | 95 (104) |
|  | 11. To bring my genuine self to a training, I need to know the correct answer for all the questions the nurses ask. | NMS | 80 (40) | 76 (38) |
|  |  | NM | 28 (31) | 31 (34) |
| **Episode 4- Professor Agni Attacks the Pre-brief** | 12. The pre-brief is part of making a safe learning space | NMS | 96 (48) | 90 (45) |
|  |  | NM | 95 (105) | 86 (95) |
|  | 13. A participant can ask the facilitator for clinical help at any time during a simulation | NMS | 92 (46) | 88 (44) |
|  |  | NM | 91 (100) | 81 (89) |
|  | 14. Identify the 4 components of the pre-brief | NMS | 90 (45) | 80 (40) |
|  |  | NM | 85 (94) | 53 (58) |
| **Episode 5- Super Divya Defends the Pre-brief** | 15. The facilitator spray is an imaginary tool that can be used to: | NMS | 52 (26) | 48 (24) |
|  |  | NM | 37 (41) | 28 (31) |
| **Episode 6- Teamwork and communication Part 1** | 16. The learning lens is a helpful tool when the power/electricity goes off | NMS | 96 (48) | 78 (39) |
|  |  | NM | 60 (66) | 64 (70) |
|  | 17. Different communication techniques such as two challenge rule or check back could prevent you from making a clinical error during an emergency | NMS | 100 (50) | 98 (49) |
|  |  | NM | 91 (100) | 95 (104) |
|  | 18. She arrives at the facility with her husband and was taken to the triage room. What is the very first thing you should do when you meet Ruchi? | NMS | 90 (45) | 96 (48) |
|  |  | NM | 78 (86) | 75 (83) |
| **Episode 7- Teamwork and communication part 2** | 19. What does the communication technique SBAR stand for? | NMS | 98 (49) | 94 (47) |
|  |  | NM | 92 (101) | 90 (99) |
|  | 20. Provider 2 arrives to help Provider 1 with neonatal resuscitation. Provider 1 says, “Priya is gravida 2, para 1, with four antenatal care visits. She has had a six-hour labor. Her baby was just born and her husband is in the waiting area. Did Provider 1 demonstrate all four components of SBAR? | NMS | 86 (43) | 92 (46) |
|  |  | NM | 81 (89) | 66 (73) |
|  | 21. The fluster bugs that Professor Agni unleashed were meant to... | NMS | 88 (44) | 84 (42) |
|  |  | NM | 86 (95) | 86 (95) |
|  | 22. During a simulation there is a lot of action going on at the same time. How do you focus on the important moments? | NMS | 74 (37) | 80 (40) |
|  |  | NM | 71 (78) | 56 (62) |
| **Episode 8- Introducing the Debrief** | 23. When should a facilitator conduct a debrief huddle? | NMS | 80 (40) | 86 (43) |
|  |  | NM | 81 (89) | 85 (94) |
|  | 24. The debrief huddle helps in: | NMS | 90 (45) | 82 (41) |
|  |  | NM | 74 (81) | 72 (79) |
|  | 25. What should a simulation facilitator do during the release moment? | NMS | 50 (25) | 32 (16) |
|  |  | NM | 40 (44) | 34 (37) |
|  | 26. Empathy goggles help a facilitator identify the nurses who know the right answers during the debrief. | NMS | 84 (42) | 90 (45) |
|  |  | NM | 68 (75) | 71 (78) |
| **Episode 9- Super Divya decodes the Debrief** | 27. Why is the analysis phase of a debrief the longest? | NMS | 90 (45) | 96 (48) |
|  |  | NM | 89 (98) | 90 (99) |
|  | 28. What happens when a facilitator lets their ego get in the way of debriefing? | NMS | 4 (2) | 76 (38) |
|  |  | NM | 65 (72) | 65 (71) |
|  | 29. What should the participants do during the application phase? | NMS | 80 (40) | 76 (38) |
|  |  | NM | 49 (54) | 45 (50) |
|  | 30. How does curiosity help a facilitator? | NMS | 90 (45) | 92 (46) |
|  |  | NM | 71 (78) | 82 (90) |
